# Adult gonorrhea, chlamydia and syphilis prevalence, incidence, treatment and syndromic case reporting in South Africa: Estimates using the Spectrum-STI model, 1990-2017

**DOI:** 10.1371/journal.pone.0205863

**Published:** 2018-10-15

**Authors:** Ranmini S. Kularatne, Ronelle Niit, Jane Rowley, Tendesayi Kufa-Chakezha, Remco P. H. Peters, Melanie M. Taylor, Leigh F. Johnson, Eline L. Korenromp

**Affiliations:** 1 Centre for HIV & STI, National Institute for Communicable Diseases, Johannesburg, South Africa; 2 Department of Clinical Microbiology & Infectious Diseases, Faculty of Health Sciences, University of the Witwatersrand, Johannesburg, South Africa; 3 Health Information Systems Programme, Pretoria, South Africa; 4 Independent consultant, London, United Kingdom; 5 Anova Health Institute, Johannesburg, South Africa; 6 Department of Medical Microbiology, Faculty of Health Sciences, University of Pretoria, Pretoria, South Africa; 7 World Health Organization, Department of Reproductive Health and Research, Geneva, Switzerland; 8 USA Centers for Disease Control and Prevention, Division of STD Prevention, Atlanta, Georgia, United States of America; 9 University of Cape Town, Centre for Infectious Disease Epidemiology and Research, Cape Town, South Africa; 10 Avenir Health, Geneva, Switzerland; US Centers for Disease Control and Prevention, Dengue Branch, PUERTO RICO

## Abstract

**Objectives:**

To estimate trends in prevalence and incidence of syphilis, gonorrhea and chlamydia in adult men and women in South Africa.

**Methods:**

The Spectrum-STI tool estimated trends in prevalence and incidence of active syphilis, gonorrhea and chlamydia, fitting South African prevalence data. Results were used, alongside programmatic surveillance data, to estimate trends in incident gonorrhea cases resistant to first-line treatment, and the reporting gap of symptomatic male gonorrhea and chlamydia cases treated but not reported as cases of urethritis syndrome.

**Results:**

In 2017 adult (15–49 years) the estimated female and male prevalences for syphilis were 0.50% (95% CI: 0.32–0.80%) and 0.97% (0.19–2.28%), for gonorrhea 6.6% (3.8–10.8%) and 3.5% (1.7–6.1%), and for chlamydia 14.7% (9.9–21%) and 6.0% (3.8–10.4%), respectively. Between 1990 and 2017 the estimated prevalence of syphilis declined steadily in women and men, probably in part reflecting improved treatment coverage. For gonorrhea and chlamydia, estimated prevalence and incidence showed no consistent time trend in either women or men. Despite growing annual numbers of gonorrhea cases − reflecting population growth − the estimated number of first line treatment-resistant gonorrhea cases did not increase between 2008 and 2017, owing to changes in first-line antimicrobial treatment regimens for gonorrhea in 2008 and 2014/5. Case reporting completeness among treated male urethritis syndrome episodes was estimated at 10–28% in 2017.

**Conclusion:**

South Africa continues to suffer a high STI burden. Improvements in access and quality of maternal, STI and HIV health care services likely contributed to the decline in syphilis prevalence. The lack of any decline in gonorrhea and chlamydia prevalence highlights the need to enhance STI services beyond clinic-based syndromic case management, to reinvigorate primary STI and HIV prevention and, especially for women, to screen for asymptomatic infections.

## Introduction

South Africa has some of the highest rates of sexually transmitted infections (STIs) in the world and the government views them as a major public health problem. In 2007 the South African government introduced its first national strategic plan (NSP) for HIV and STIs and its latest plan for 2017–2022 includes STI interventions and targets [1]. STIs in South Africa have been managed syndromically using standardized national syndromic management guidelines since the mid-1990s [2]. Key strategies directed at preventing STIs include increasing access to sexual and reproductive health services including promotion of condom use, conducting prevention activities in non-traditional outlets, screening for syphilis in antenatal care, and implementing a comprehensive national social and behavioural change communication strategy with a focus on key populations.

STI surveillance in South Africa is focused on Male Urethritis Syndrome (MUS). All Primary Health Clinics (PHCs) are required to report monthly to the National Indicator Dataset (NIDS) of the South African District Health Information System [3]. The National Institute of Communicable Diseases (NICD) also undertakes etiological surveillance of STI syndromes annually at sentinel PHCs in Gauteng Province and biennially in other provinces [4,5], to validate and update existing STI syndromic management guidelines. In 2008 monitoring of antimicrobial resistance in culture isolates of *Neisseria gonorrheae*, the predominant cause of MUS [4,5] was introduced and South Africa is a participating country in the World Health Organization (WHO)’s Gonococcal Antimicrobial Surveillance Programme (GASP) [6].

Data on the prevalence of syphilis in adults in South Africa is collected regularly through sentinel surveys of women attending antenatal clinics (ANCs) [7,8] and integrated biological and behavioural surveys in key populations [9]. In addition, in 2009 South Africa started collecting test results from routine programmatic screening in ANC. For gonorrhea and chlamydia these type of data are not available; prevalence data come from research projects and clinical trials.

South Africa’s STI survey and surveillance data have been used to estimate national prevalence patterns and distributions using dynamic STI transmission models. A first round used STI prevalence data collected up to 2002 [10], and the most recent round data up to 2011 [11,12]. The results suggested that syphilis and gonorrhea prevalence began to decline in the late 1990s. Much of this decline was attributed to increased condom use in response to HIV communication programmes and the introduction of syndromic management protocols; in contrast, chlamydia prevalence did not change [13]. However, South African STI prevalence data have not been systematically reviewed after 2005, and dynamic models have not been updated to incorporate more recent STI prevalence data. STI prevalence trends estimated by transmission dynamic models are useful tools for looking at how STIs are spread, but the results reflect the underlying assumptions about trends in sexual behaviors, population networks and STI treatment, and may not reflect true trends if the assumptions are unrealistic. There is thus a need to assess STI prevalence trends using alternative (static or non-dynamic) models that require fewer assumptions. There is also a need for model-based estimates that reflect more recent STI prevalence data.

High rates of STIs estimated historically, and high rates of antimicrobial resistant gonorrhea detected in ongoing surveillance, prompted South Africa to undertake a national STI estimation effort including estimation of trends in the population burden of antimicrobial resistant gonorrhea. A national workshop as held in March 2018, that brought together experts and stakeholders from national STI, HIV and reproductive health programs and academia to collate and synthesize recent national STI data. It used the epidemiological modelling tool Spectrum-STI [14–18]. Spectrum-STI (http://avenirhealth.org/software-spectrum.php) is a statistical trend estimation model developed by Avenir Health, with support from the World Health Organization (WHO), for use by national health officials to synthesize STI surveillance data and generate national trend estimates. For South Africa’s use, Spectrum-STI was adapted to additionally assess population-based trends in resistant gonorrhea cases using surveillance-based trends in antimicrobial resistance.

This paper documents the results from the South Africa Spectrum-STI estimation. The main outputs are adult prevalence and incidence trends for active syphilis, gonorrhea and chlamydia in adult women and men, and trends in the number of new cases of antibiotic-resistant gonorrhea in men. In addition, we compare estimated numbers of symptomatic and treated gonorrhea and chlamydia cases with numbers of MUS cases reported in South Africa, to gauge the completeness of routine case reporting.

## Methods

No individual-patient data or records were used. We only used already published, population-aggregated data sets, which were all fully anonymized.

### Syphilis: Prevalence and incidence

Prevalence data were identified from studies conducted between 1985 and 2017 in populations representative of the general adult (15–49 year-old) population in South Africa (S1 File) [11,12]. Eligible study populations included pregnant women in ANC care (routine screening or surveys), women attending family planning clinics, and women and men sampled in household surveys and other community-based studies. For women, all data from ANC and non-ANC women were pooled, assuming no systematic prevalence differences between them, as in the 2012 WHO methodology [19] and supported by a recent global meta-analysis of risk factors for adult syphilis [20].

Data were adjusted for diagnostic test performance to reflect ‘active’ syphilis, defined as concurrent positivity on both non-treponemal Rapid Plasma Reagin (RPR) and treponemal tests (e.g. the Treponema pallidum haemagglutination assay (TPHA)[21], using the same assumptions as other Spectrum estimates [22]. Prevalence data from studies that used both a treponemal and non-treponemal test were not adjusted. Prevalence values for studies using rapid treponemal tests were multiplied by 0.70, studies using only treponemal or only non-treponemal tests without confirmatory test were multiplied by 0.53, and studies where the diagnostic test was unknown by 0.75. Each adjustment multiplier was assumed to have a standard error of ±25%. In addition, each syphilis data point data increased by 10% to account for under-sampling of high-risk populations [19], an adjustment in keeping with key group prevalences from Integrated Bio-Behavioural Surveys in South Africa [9,23,24], and estimated population sizes [9]. No adjustments were made for age or location. The adjusted data points were each assigned a weight to reflect national coverage and representativeness. Nationally representative data were assigned a weight of 100% whilst data representative of smaller areas, or from a subset of sentinel surveillance sites were weighted accordingly [22] (S1 File).

The Spectrum-STI model fitted the prevalence data by segmented polynomials regression [25,26] applying a second-order spline curve. Both the number and positions of the knots were estimated, and the Akaike Information Criterion was used for model selection’ [22]. Estimated prevalence was constrained to be within 0–20% [22]. Uncertainty bounds were generated using bootstrapping, with 10,000 replications [22].

Annual incidence was derived by dividing the estimated prevalence by the average duration of infection [19] using the 2012 WHO average durations of infection values for countries with low and intermediate treatment access (S1 File). In the default scenario the proportion of symptomatic episodes treated was set at 35% (the WHO estimates for countries with low treatment access [19]) between 1990 and 1997 and then assumed to increase linearly to 60% in 2006 and to remain constant after this. The increase between 1997 and 2006 was included to reflecting the roll-out of syndromic management [2,12,13] in the mid-1990s [27].

### Gonorrhea and chlamydia: Prevalence and incidence

Prevalence data were identified from studies conducted between 1990 and 2017 in female and male populations representative of the general adult 15–49 year-old population in South Africa (S1 File) [11,12]. Eligible populations included pregnant women attending ANC, women and men attending family planning clinics or PHCs, and adult women and men tested as part of household or community surveys. Eligible diagnostic tests were Nuclear Acid Amplification Tests (e.g. Polymerase Chain Reaction and Ligase Chain Reaction) and culture.

Prevalence data from each study were adjusted for diagnostic test performance [19,28,29], geographical location and age, using the same approach and parameters as the WHO’s 2012 STI estimates [19]. Diagnostic tests were adjusted using fixed sensitivities and specificities, and studies conducted in exclusively rural or urban sites were converted into a national prevalence by applying a rural-to-urban ratio of 0.9, and South Africa’s annual urban and rural population sizes [30]. Each prevalence data point was also increased by 10% to account for the contribution of higher-risk populations [19], an adjustment in keeping with key group prevalences measured in South Africa [24,31], and estimated population sizes [9].

For chlamydia in women, prevalence data were adjusted for age reflecting the decline with age of chlamydia prevalence observed in recent surveys of women in South Africa [32,33], the United Kingdom [34] and the United States of America [35] and among women in South Africa presenting to clinics for vaginal discharge syndrome (VDS) [5,36]. This marked age pattern reflects acquired immunity against reinfection with chlamydia [37]. In order to obtain prevalence estimates for the 15–49 age range, prevalence data from studies that only sampled younger populations (15–24 years of age) prevalence estimates were therefore multiplied by 0.60, and studies of exclusively older populations (25 and over) by 1.39. In the absence of similar data for men, we did not assume an age pattern for chlamydia in men. No adjustments were made for age for gonorrhea in either men or women, lacking clear evidence.

Each data point was assigned a weight to reflect its representativeness. Studies that used NAAT diagnostic tests in populations representative of the overall adult population were assigned a weight of 1.0; all other studies were assigned a weight of 0.10 reflecting uncertainties in diagnostic tests, age adjustors and the representativeness of the population sampled.

Spectrum-STI was used to generate estimates for gonorrhea and chlamydia in women and in men separately. A moving average time trend curve was fitted through the adjusted and weighted prevalence data, as there was insufficient data to use a segmented polynomials regression. Uncertainty bounds were calculated by bootstrapping (10,000 replications)[14].

Trends in annual incidence for both infections were estimated using the same approach as syphilis, and the same default assumptions for treatment coverage. Duration estimates were based on the WHO 2012 assumptions with one change: the probability of a man with chlamydia developing symptoms was set at the value used in South African transmission dynamic models, 0.33 [10,38], rather than the WHO global value of 0.54.

### Male urethritis syndrome: Case load and reporting completeness in 2017

Spectrum-estimated numbers of symptomatic cases of gonorrhea and chlamydia in 2017, and the subsets of these symptomatic cases treated, were compared to the national-level MUS case reports for 2017 collated by the South African District Health Management Information System (S2 File). Based on etiological data on the causes of MUS from South Africa [5,36,39,40] we have assumed 76% of MUS cases are due to gonorrhea and 24% to chlamydia, considering that 5% of MUS cases do not have a gonococcal and/or chlamydial etiology, but that the etiological studies found considerable rates of dual gonococcal plus chlamydia etiologies.

### Gonorrhea: Antibiotic-resistance

The Spectrum-estimated incident cases in men were multiplied by antibiotic resistance prevalence estimates from the NICD gonococcal antimicrobial resistance surveillance among men with MUS in four provinces [4,5,41,42] to estimate the incident numbers of antibiotic-resistant gonorrhea in men from 2007 to 2017.

### Sensitivity analysis

Univariate sensitivity analyses looked at relaxing the data inclusion criteria to include blood donors, who were excluded from the default estimation because of their likely non-representativeness of overall populations, as transfusion services often exclude donors with self-reported risks or observed infections (syphilis only); removing the representativeness weight adjustment (all three infections); and assuming no change in treatment coverage over time (all three infections). More general assumptions of the Spectrum-STI methodology have been addressed in sensitivity analyses in earlier publications [14,18,22].

## Results

### Syphilis

**Fig 1:**
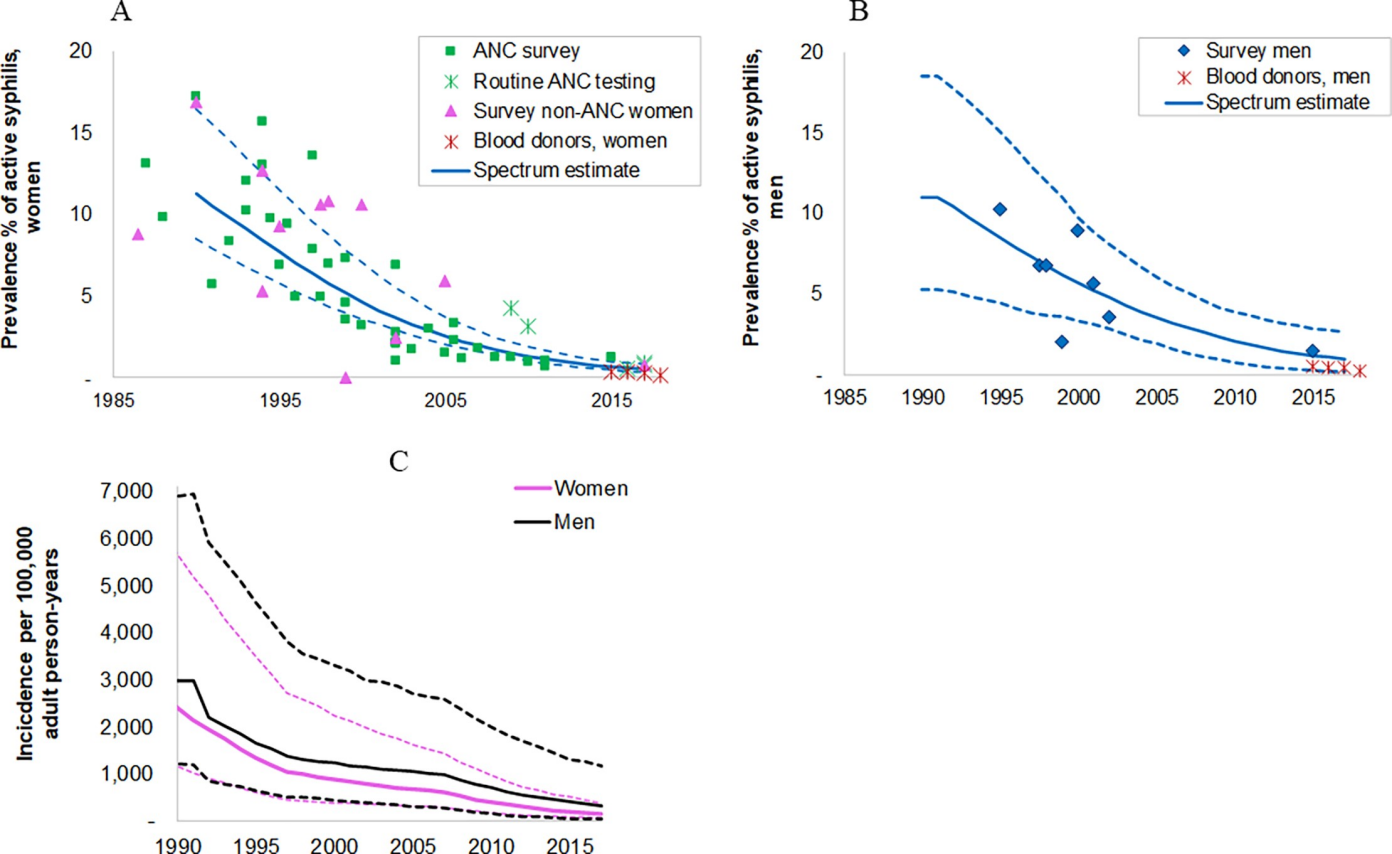
Estimated trends in active syphilis in South African adults, 15–49 years. **(a) prevalence in women; (b) prevalence in men; (c) incidence in women and men.** Prevalence data shown have been adjusted for diagnostic test performance and under sampling of high-risk populations, as described in the methods and detailed in S1 File. Solid lines are the best estimate; dotted lines are 95% Confidence Intervals. Blood donor data are shown, but these were not used in the default scenario, they were only used in the sensitivity analysis.

A total of 44 prevalence data points from pregnant women attending ANC were identified from 1985 and later: 5 data points from nation-wide routine ANC screening, and 39 national and sub-national sentinel surveys and prevalence studies (S2 File). In addition, 11 prevalence measurements from non-pregnant women (1990–2017) were also identified that met the study inclusion criteria. Fig 1A shows the adjusted prevalence data and the Spectrum estimation for women.

The estimated prevalence in 2017 was 0.50% (0.32–0.80%) (Table 1), a marked decrease from the 1990 estimate of 11.3% (8.5–16.4%).

**Table 1:** Spectrum-estimated STI prevalence, incidence rates and incident case numbers, in South African women and men 15–49 years in 2017.

STI	Metric	Women		Men	
		Point estimate	95% CI	Point estimate	95% CI
**Active syphilis**	Prevalence	0.50%	0.32% to 0.80%	0.97%	0.19% to 2.38%
	Incidence rate per 100 000 adult person-years	153	65 to 414	316	34 to 1,162
	New incident cases	23,175	9,900 to 62,500	47,500	5,100 to 173,000
**Gonorrhea**	Prevalence	6.6%	3.8% to 10.8%	3.5%	1.7% to 6.1%
	Incidence rate per 100 000 adult person-years	16,100	7,700–38,900	14,200	6,900–24,700
	New incident cases	2.3 million	1.1–5.0 million	2.2 million	1.1–3.8 million
**Chlamydia**	Prevalence	14.7%	9.9% to 21%	6.0%	3.8 to 10.4%
	Incidence rate per 100 000 adult person-years	14,400	8,000–33,100	24,900	14,100–40,800
	New incident cases	1.9 million	1.1–3.8 million	3.9 million	2.2–6.3 million

95% CI = 95% confidence interval.

Fig 1B shows the corresponding prevalence estimates for men. In men, owing to a lack of data pre-1995 the prevalence from 1990 to 1995 was fixed at the 1995 level. Between 1995 and 2017 prevalence fell from 8.2% (4.1–13.2) to 0.97 (0.19–2.38). These estimates were based on the 8 data points identified in male general populations.

The number of new cases in 2017 (Table 1) was estimated to be 23,175 (9,900–62,500) in women and 475,000 (5,100–173,000) in men. The annual incidence (Fig 1C) mirrored the decline in prevalence, with incidence in 2017 9-fold lower in women and 5-fold lower in men than in 1995. The decline in incidence was less than the decline in prevalence reflecting a decrease in the average duration of infection between 1997 and 2006 owing to our assumption that treatment coverage increased over this period.

### Gonorrhea

**Fig 2:**
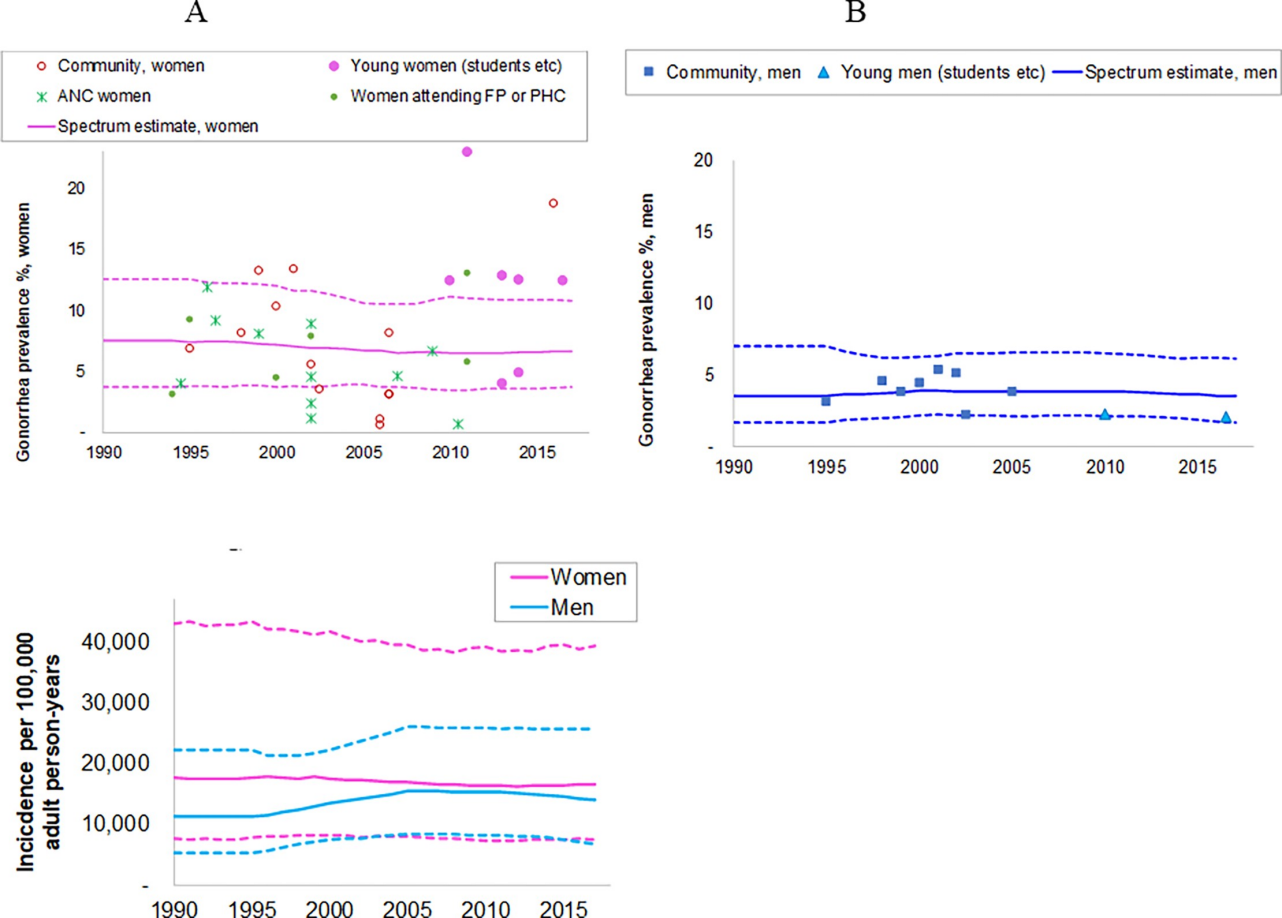
**Estimated trends in gonorrhea in South African adults, 15–49 years: (a) prevalence in women; (b) prevalence in men; (c) incidence in women and men.** Prevalence data shown have been adjusted for diagnostic test performance, urban/rural location, and under sampling of high-risk populations, as described in the methods and detailed in S1 File. Solid lines are best estimates; dotted lines are 95% Confidence Intervals. ANC = antenatal care clinic attendees, FP = family planning clinic attendees; PHC = Primary health clinic attendees.

Forty-nine eligible prevalence measurements (36 female and 10 male) were identified between 1990 and 2017 (S1 File, Fig 2A and 2B). The data represented both urban and rural locations in all nine provinces.

The Spectrum estimated gonorrhea prevalence in 2017 was 6.6% (95% CI: 3.8–10.8%) in women and 3.5% (1.7–6.1%) in men (Fig 2A and 2B). Prevalence over 1990–2017 was stable in both sexes. The time trend in incidence of new infections mirrored this (Fig 2C), except for a slight increase between 1997 and 2006 in men when treatment coverage was assumed to increase to reflect the roll-out of syndromic treatment.

Spectrum estimated there were 2.3 (1.1–5.0) million and 2.2 (1.1–3.8) million new gonorrhea cases (i.e. episodes) in women and men in 2017 (Table 1). The case incidence rate and number of new cases were similar in men and women, despite the prevalence being higher in women, reflecting the longer average duration of infection in women.

### Chlamydia

For chlamydia, 46 prevalence data points (35 female and 11 male) were identified from the period 1994 to 2017 from urban and rural locations across all 9 provinces of South Africa.

**Fig 3:**
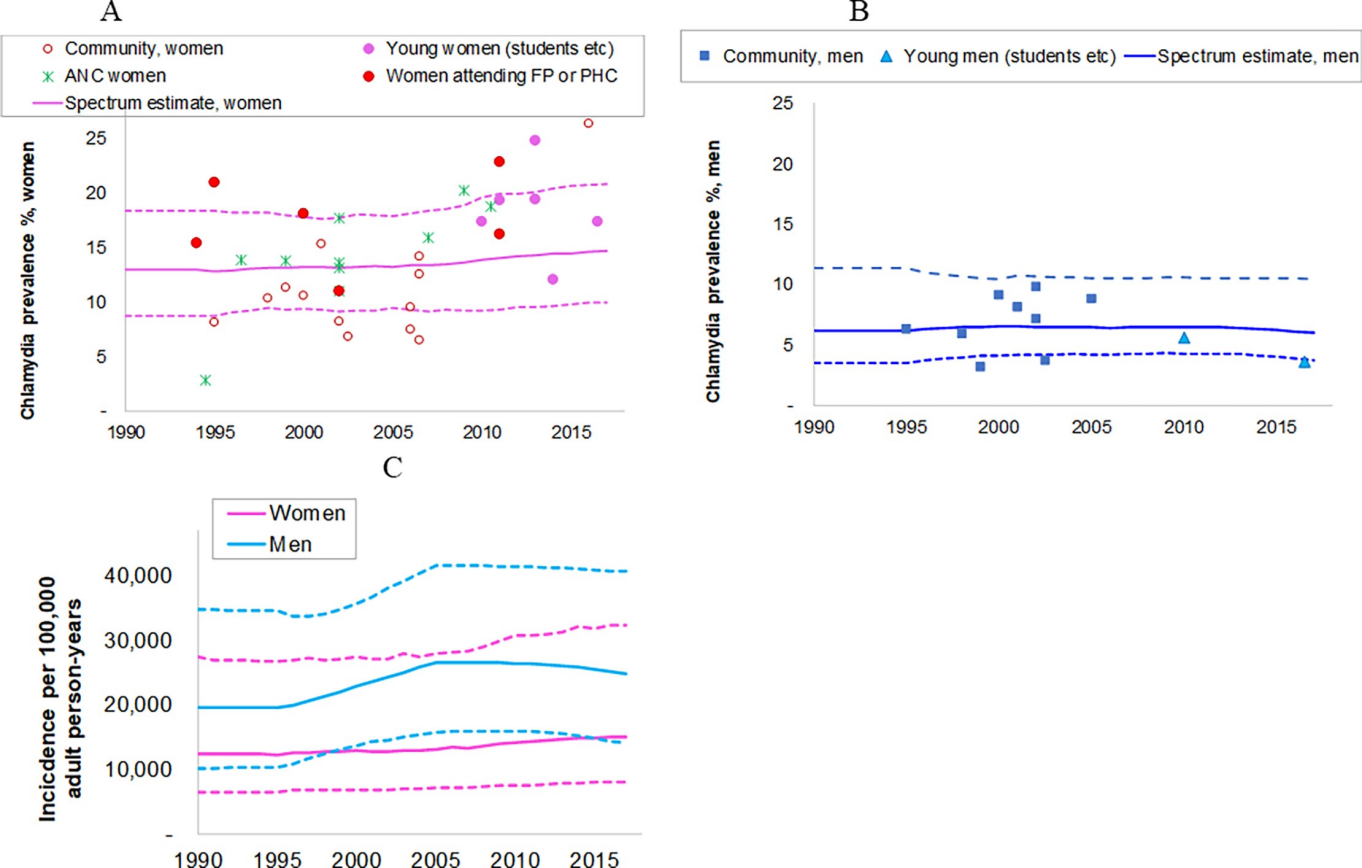
**Estimated trends in chlamydia in South African adults, 15–49 years: (a) prevalence in women; (b) prevalence in men; (c) incidence in women and men.** Prevalence data shown have been adjusted for diagnostic test performance, urban/rural location, and under sampling of high-risk populations, as described in the methods and detailed in S1 File. Solid lines are best estimates; dotted lines are 95% Confidence Intervals. ANC = antenatal care clinic attendees, FP = family planning clinic attendees; PHC = Primary health clinic attendees.

Chlamydia prevalence in 2017 was estimated at 14.7% (9.9–21%) in women and 6.0% (3.8–10.4%) in men (Fig 3A & 3B). The time trend in prevalence over 1990–2017 was stable for both sexes and the incidence trend mirrored this apart from an increase over 1997 to 2006 when treatment coverage was assumed to increase.

Spectrum estimated that there were 1.9 (1.1–3.8) million new chlamydia cases in women and 3.9 (2.2–6.3) million new infections in men in 2017 (Table 1). The chlamydia case incidence rate and number of new cases were higher in men than in women, despite chlamydia prevalence being higher in women, reflecting the longer average duration of infection in women.

### Gonorrhea: Antibiotic-resistance

**Fig 4:**
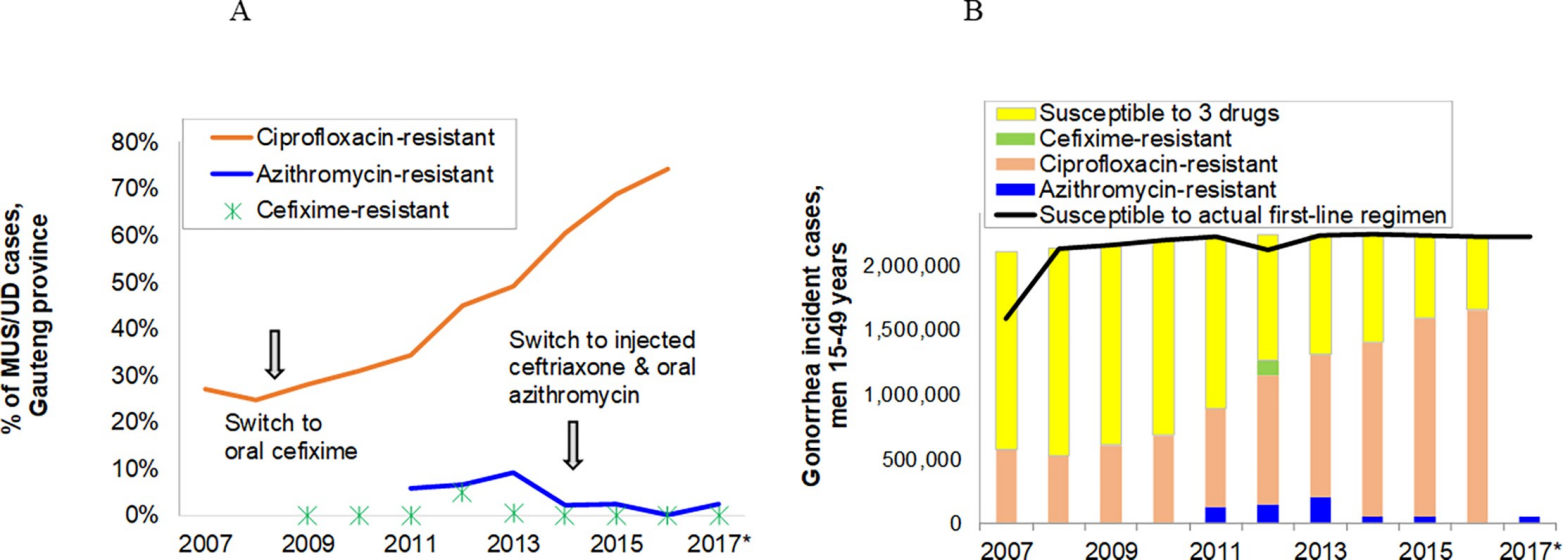
**Spectrum estimates of antibiotic resistant gonorrhea, South Africa: (a) Proportion of gonorrhea cases among men presenting to PHCs with male urethritis syndrome in national sentinel surveillance; (b) estimated annual incident gonorrhea cases resistant and susceptible to the 3 first-line antibiotic treatments shown in Fig 4A**. • Ciprofloxacin: Resistance data shown includes intermediate resistance (Minimum Inhibitory Concentration (MIC) = 0.12–0.50 μg/mL, and full resistance (MIC of ≥1 μg/mL) ([68]. • Azithromycin: Resistance as estimated and shown is defined as Intermediate resistance, i.e. MIC = 0.50 μg/mL [69]; • Cefixime: Resistance as estimated and shown is defined as MIC = 0.25 μg/mL [69]. Annual surveillance in Gauteng province did not detect any consistent, progressive and sustained increase by year in MIC_50_ (minimum antimicrobial concentration needed to inhibit 50% of isolates) & MIC_90_ (minimum antimicrobial concentration needed to inhibit 90% of isolates). • Ceftriaxone: Resistance, defined as MIC = 0.25 μg/mL (EUCAST 2018; version 8.0), was detected in 2/324 (0.6%) of cases surveyed in 2009, but in 0% of cases in 2008 and 2010–2017 (number of cases ranged from 128 to 338); and no significant increase in the MIC over time was detected. • In 2017 ciprofloxacin resistance was not tested by NICD; cefixime (CXM), ceftriaxone were tested and no resistance detected to the extended-spectrum cephalosporins. The prevalence of intermediate resistance to azithromycin was 1.8%. Sources: National Institute of Communicable Diseases [4,5,41,42].

Fig 4A shows the evolution of antibiotic resistance for gonorrhea, based on sentinel surveillance data from 2007 to 2017. In response to antimicrobial resistance trends, South Africa changed its first-line treatment regimen for MUS in 2008 from ciprofloxacin (used since 1993) to oral cefixime, and in 2014/15 from oral cefixime to dual injectable single-dose ceftriaxone & oral azithromycin [2], in accordance with WHO recommendations [43]. Dual antimicrobial cover for gonorrhoea is a strategy designed to curtail the emergence of extensively-drug resistant *N*. *gonorrhoeae* that is resistant to the extended-spectrum cephalosporins. This gained additional importance in 2012 with the first reports of cefixime resistance and resultant treatment failure for male urethritis in South Africa in two MSM [42], in recognition of the fact that there is a paucity of gonococcal antimicrobial resistance surveillance data from key population groups in the country.

The observed antibiotic resistance prevalence estimates from four provinces were applied to the population-level male gonorrhea prevalence estimates for 2007 to 2017. In each year the majority of cases were susceptible to the prevailing year’s first-line treatment (Fig 4B). The continued increase in the prevalence of *N*. *gonorrheae* resistance to ciprofloxacin after its withdrawal from use in STI syndromic management algorithms may reflect ongoing antimicrobial selection pressure arising from its use for other indications or its continued use by public and private STI treatment providers during shortages or stock-outs of the first line treatment [44]. In addition, there may be a fitness benefit conferred by ciprofloxacin-resistance associated mutations [45].

### Male urethritis: Case load and reporting completeness

In 2017, of the 2.2 million Spectrum-estimated gonorrhea cases in adult men, 1.4 million were symptomatic and 850,000 million were treated (Table 2). For chlamydia Spectrum estimated 3.9 million cases in adult men in 2017 of which 1.3 million were symptomatic and 765,000 were treated. Using the gonorrhea estimates and assuming 74% of symptomatic MUS cases are due to gonorrhea the estimated number of treated MUS cases in 2017 was 1.1 million. A similar calculation for chlamydia, assuming 26% of symptomatic cases are due to chlamydia, comes to 3.2 million treated MUS cases.

**Table 2:** Incident cases of gonorrhea, chlamydia and male urethritis syndrome, and the subset who are symptomatic, treated and reported, and the implied MUS case reporting completeness in men 15–49 years in South Africa, 2017.

	Gonorrhea	Chlamydia	Source & assumptions
Estimated incident cases	2.21 million	3.87 million	Spectrum-STI estimate
Estimated symptomatic cases	1.42 million	1.28 million	Assumed 64% of gonorrhea [19] and 33% of chlamydial infections [10] symptomatic.
Estimated symptomatic cases of gonorrhea or chlamydia treated	850,000	765,000	Assumed 60% of symptomatic cases treated [19].
Estimated symptomatic MUS cases (all etiologies) treated	1.12 million	3.19 million	Assumed 74% of MUS cases due to gonorrhea and 26% due to chlamydia [5,36,39,40]. The 1.12 million and 3.11 million are two alternative, mutually exclusive estimates.
Actual new MUS case reports	310,921		
Reporting completeness among treated MUS cases	310,921 / 1.12 million = 28%	310,921 / 3.19 million = 10%	The 28% and the 10% are two alternative, mutually exclusive estimates.

Abbreviations: MUS = Male Urethritis Syndrome.

The actual number of MUS cases reported by the South African STI program from public clinics was 310,921 (S2 File). Comparing the actual MUS cases to the Spectrum estimates of MUS cases treated suggests that case reporting completeness, among treated MUS cases, is somewhere between 10% and 28%.

### Sensitivity analyses

Table 3 summarizes the results of the sensitivity analyses. Relaxing the study eligibility criteria for syphilis to include national blood donor screening data from 2015–2018 (S1 File) reduced the estimated prevalence of syphilis in women from 0.50% to 0.38% and in men from 0.97% to 0.35%. Including blood donor data had less of an impact on the prevalence in women than men owing to the large number of data points for women from ANC surveys and ANC-based routine screening. Including blood donors markedly reduced the male-to-female prevalence ratio, from almost 1.9:1 to 0.92:1, a figure much closer to the 1:1 ratio found in studies within South Africa [46], across East and Southern Africa [47–49], and elsewhere [20].

**Table 3:** Sensitivity analysis–effect of varying (selected) assumptions and values, on estimated national STI prevalence and numbers of new infections for adult men or women (15–49 years) in South Africa, in 2017.

Scenario & STI	Syphilis		Gonorrhea		Chlamydia		Syphilis	Gonorrhea	Chlamydia
Metric	Prevalence						Incident cases		
Sex	Women	Men	Women	Men	Women	Men	Men	Men	Men
Default scenario	0.50% (0.32–0.80)	0.97% (0.19–2.38)	6.6% (3.8–10.8)	3.5% (1.7–6.1)	14.7% (9.9–21)	6.0% (3.8–10.6)	47,500 (5,100–173,000)	2.2 (1.1–3.8) million	3.9 (2.2–6.3) million
Including sex-specific blood donor screening data from 2015 to 2018 (see S1 File)	0.38% (0.25–0.69)	0.35% (0.19–0.52)	−	−	−	−	16,000 (4,900–49,800)	−	−
All gonorrhea & chlamydia data points assigned the same statistical weight	−	−	8.4% (4.7–13.4)	2.7% (1.1–5.6)	17.3% (11.7–23.9)	5.1% (2.4–9.3)	−	1.8 (0.70–3.2) million	3.2 (1.5–6.5) million
Scenario 4. All 3 STIs: Assume that treatment coverage stays at 35% throughout 2017 (instead of improving to 65%; for syphilis in M&F and for NG and CT in M)	−	−	−	−	−	−	39,100 (4,200–142,700)	1.8 (0.75–3.1) million	3.4 (2.2–5.9) million

‘−‘ means: Not assessed, because the outcome was not affected by the scenario.

Assigning the same weight to all studies increased the prevalence in 2017 of gonorrhea and chlamydia in women from 6.6% to 8.4% and from 14.7% to 17.3% (Table 3) reflecting the prevalence in two studies from 2017 that documented high prevalences for both infections in young women [50] and in a rural area with relatively poor access to health care [51]. For men, the prevalence of gonorrhea decreased from 3.5% to 2.7% and of chlamydia from 6.0% to 5.1% reflecting data from a study in young men [50]. Gonorrhea and chlamydia incidence estimates in men decreased proportionally; relative to the incidence estimates in this scenario, we would have estimated a correspondingly higher reporting completeness of routine national MUS case reporting: 11–35% of symptomatic treated cases, compared to 10–28% in the default estimates. The weighting did not materially change results for syphilis (Table 3).

Changing the treatment coverage assumptions such that treatment coverage had not improved between 1997 and 2006 and instead remained at the 1997 level reduced the estimated number of new cases in men in 2017 from 45,000 to 37,100 for syphilis, from 2.2 million to 1.8 million for gonorrhea, and from 3.8 to 3.4 million for chlamydia. The lower number of new cases in 2017 in this scenario reflects the assumed longer average duration of infection (constant throughout 1990–2017), driven by a lower (constant) treatment coverage throughout 2017.

## Discussion

The Spectrum-STI estimates confirm the high burden of STIs in South Africa. The 2017 adult prevalence estimates of 6.6% and 3.4% for gonorrhea and 14.7% and 6.0% for chlamydia in women and men, respectively, are among the highest in the world [19] and the estimated prevalence of active syphilis of above 0.5% in both men and women remains high, too. Whilst the prevalence estimates of syphilis have fallen by more than an order of magnitude since 1990, gonorrhea and chlamydia prevalence have remained almost unchanged despite South Africa’s large investment in HIV and STI prevention and treatment efforts. The Spectrum estimates are comparable to those from the earlier South African transmission dynamic modeling exercises for 1990 to 2006 [10,13], which also estimated declines in syphilis but little change in gonorrhea or chlamydia.

STI case reports tell a slightly different story: data from the national reporting system show a steady decline in Genital Ulcer Syndrome (GUS), MUS and VDS between 2005 and 2016 from sentinel sites and national PHC clinics (S2 File). As the main causes of MUS are gonorrhoea and chlamydia and among sexually-transmitted aetiologies of VDS, chlamydia is predominant, assuming no changes in access to health care or in the reporting systems, this would suggest that the incidence of both infections had fallen. The discrepancy between the case reports and the estimates from both the Spectrum statistical model and transmission dynamic models highlight the need for renewed efforts to improve access to and reporting by health services, and collect independent population-level (non-clinic-based) data for these two infections on a regular basis. STI case reports are a useful piece of information, but as many other middle-income countries [17,52,53] have noted are not, on their own, sufficient indicators for monitoring STI progress or impact, or as programmatic goals.

The decline in the prevalence of syphilis is consistent with the decline in the proportion of genital ulcer syndromes (GUS) attributed to syphilis among mineworkers in Carletonville (Gauteng province) over 1986–1998 [54], and data from sub-national [39] and national syndrome etiological surveillance since 2007 [5,36,40]. It may reflect improvements in clinical STI services, including the expansion of syndromic management for GUS and partner notification in the public sector [55], rolling out a national ANC-based syphilis screening program between 1998 and 2013, screening for syphilis as part of the HIV program, and since 2009 offering (free) medical male circumcision. However, the decline may also have been driven by selective HIV/AIDS mortality among higher-risk individuals [56].

The decrease in syphilis in adult females has driven a parallel decline in congenital syphilis (CS) [57] and, whilst South Africa’s CS case rate is still above the WHO elimination threshold of 50 per 100,000 live births, progress is being made towards eliminating mother-to-child transmission of CS, a key objective of the NSP [1]. Expansion of dual syphilis/HIV testing at ANC, especially in more remote rural areas, and at PHCs for late ante-natal bookers, should help South Africa reach this target quicker.

The persistently high gonorrhea and chlamydia rates, similar to stable etiological fractions of these STIs in national etiological surveillance studies of VDS (S2 File) and MUS [5,36,39,40], indicate that the impact of primary HIV/STI prevention on gonorrhea and chlamydia has been modest. The picture for HIV is similar: epidemiological modelling of South Africa’s HIV epidemic and response indicates that whilst HIV incidence has declined from the levels reached at the end of the 1990s it remains high despite prevention efforts and increasing coverage of antiretroviral treatment [58].

Comparing the Spectrum estimates of the number of symptomatic men with gonorrhea or chlamydia with the number of MUS cases reported each year suggests that less than 28% of symptomatic cases in men (even apart from additional asymptomatic cases) are treated and reported through the NIDS. The missing cases are either not treated at all, treated in the public sector but not reported, or treated in the private sector or by informal providers and not reported. Getting a better understanding on these missing cases will help target surveillance, control and treatment efforts. There is also a need to better understand treatment and reporting in women.

Spectrum incidence estimates combined with gonococcal antimicrobial resistance prevalence data in Gauteng Province suggest that annual numbers of cases of gonorrhea resistant to first-line treatment have not increased, owing to changes made to the treatment regimens for gonorrhea in 2008 and 2014/5 (Fig 4B). As a result of effective monitoring of antimicrobial resistance and policy changes tailored according to resistance trends, the emergence and spread of extensively-drug resistant gonorrhea has not been observed.

The South African Spectrum STI estimation exercise provides information that can be used to inform STI estimation exercises in other countries. The male-to-female prevalence ratios for 2017, 0.53 for gonorrhea and 0.41 for chlamydia, are in line with earlier modeling estimates for South Africa [10–13] but are lower than the global ratios used in the WHO 2012 estimates (0.86 and 0.80) [19]. The lower male to female ratios in South Africa, compared to the global figures are in keeping with the relatively high STI burden in South Africa and the national STI syndromic management policy. Syndromic treatment is expected to treat more male than female STI patients as women are more often asymptomatic, symptoms in women are frequently non-specific, and women often have poorer access to health care services or decide not to seek care [59].

### Limitations

The Spectrum-STI South African estimates are limited by the quality and representativeness of prevalence data, and by assumptions underlying the model and those made in the absence of data. Whilst there was a sufficient base to generate time trends for the three STIs for both men and women, there is considerable uncertainty for gonorrhea and chlamydia as the data are from studies done in different populations using different study designs. Many of the prevalence data points, especially for gonorrhea and chlamydia, are from research projects and the study population may not be representative of the general population. In addition, the studies are from different age groups and we have only made age adjustments in the case of chlamydia in women and these are crude adjustments. Other limits to the data include the range of different diagnostic tests used and whilst we have corrected for diagnostic sensitivities and specificities the correction factors may not be correct. For example, our prevalence estimates for 2017 may be too high if the studies included for recent years over-sampled higher-risk populations, or for chlamydia younger populations, or if recent studies used diagnostic tests of higher sensitivity than we assumed. The lack of data also precludes any analysis at the sub-national level, which with the current non-systematic data set would risk to confound diversity in demographic, socio-economic and behavioural variations with real geographical patterns.

The present estimates focused on data from the general population and were not informed by prevalence data from high-risk groups. A new version of the Spectrum model is under development that incorporates risk groups and means that the current, crude approach of adding 10% to the general population estimate to reflect these missing populations will no longer be required.

### Recommendations

STI surveillance and control in South Africa would clearly benefit from regular, systematic prevalence screening. For surveillance, the ideal would be periodic (e.g. 3-yearly) cluster-sample surveys, in men and women, in selected urban and rural sites. Securing funding for expanded etiological STI screening will be challenging, but there are opportunities to add to existing or planned activities, such as in pregnant women, adolescent girls, young women and other high-risk women, and priority populations such as MSM [60]. In both routine health care settings and through outreach, this would ideally use point-of-care tests (POCTs) with acceptable performance characteristics (affordability, equipment-free testing and delivery of results within 30 minutes), and be accompanied by partner notification and treatment. Of commercially available POCTs for gonorrhoea and chlamydia screening, the Cepheid geneXpert CT/NG (duplex for *Neisseria gonorrhoeae* and *Chlamydia trachomatis*) has demonstrated acceptable performance in terms of sensitivity and specificity, including when used on non-invasive specimens such as first-void urine [61]. With the Cepheid Xpert MTB/Rif assay now widely implemented for tuberculosis screening across public health sector laboratories in South Africa, existing geneXpert diagnostic capacity for TB could be leveraged to include screening for asymptomatic STIs in at-risk populations, which would also enable pathogen-directed expedited partner therapy. Additionally, there is a need to reinforce the training of both public and private sector healthcare workers in correct STI syndromic management, through the use of continuing medical education activities and educational material, including visual aids. Studies evaluating STI services at both private and public healthcare facilities in South Africa have revealed that general knowledge regarding STI aetiologies and management and compliance with national treatment guidelines among healthcare workers is suboptimal [44,62], and this compromises quality of care resulting in the prescription of incorrect treatment regimens for STI syndromes.

There is also a need to strengthen case reporting in both the public and private sector if trends in case reports to be a meaningful indicator. For a start, conducting regular, periodic data quality audits at facility-level could assess the quality of existing reporting systems, and determine the degree of under-reporting. Also, MUS could be added to South Africa's Rapid Internal Performance Data Audit (RIPDA) tool [63,64], used by health facilities for internal auditing before the Auditor General evaluates the Department of Health performance by comparing data as found in facility-level source documents with data in the District Health Information System 2. Third, quarterly feedback to provinces and districts about STI indicator performance, could stimulate action. Fourth, printed booklets of the STI surveillance tools will promote consistent and frequent data collection in sentinel sites. Finally, setting case finding targets can be a helpful mechanism to promote consistent case reporting, especially when coupled with period evaluation of actual performance against those targets.

The stable high rates of gonorrhea and chlamydia highlight that syndromic case management is not sufficient to control STIs. One policy change that would make a difference is to remove the current age constraint on STI treatment among women presenting with VDS; at present a woman presenting with VDS is treated for an STI only if she is below 35 years of age, gives a history of a male partner with urethral discharge, or if she is a returning patient and reports no improvement in symptoms [2].

For gonorrhea treatment, the regularly revised treatment guidelines are adequate in view of antimicrobial resistance patterns in public-sector surveillance. However, antimicrobial resistance monitoring clearly remains a priority area–and there may be scope to extend screening for extensively-drug resistant gonorrhea to key populations such as MSM, to expand sampling to include symptomatic and asymptomatic men at both genital and extra-genital sites, and to expand resistance surveillance and prescription patterns in the private sector where ciprofloxacin continues to be prescribed [44].

South Africa’s high STI rates also underscore the national policy to not limit VMMC to HIV-negative men, but to also enable HIV-infected men, after clinical assessment and enrolment into HIV care, to receive VMMC, which may reduce their and their partners’ STI rates [65,66].

### Conclusion

The Spectrum-STI estimates, based on STI surveillance and monitoring data, highlight the high burden of STIs in South Africa. Whilst the prevalence and incidence of syphilis in adults has fallen between 1990 and 2017, the prevalence and incidence of gonorrhea and chlamydia, with high burden especially in women who are often asymptomatic, have remained almost unchanged and are high relative to other countries. Both gonorrhea and chlamydia have health consequences for the infected individual and for babies born to infected mothers, and are established co-factors for HIV transmission [67], especially in partnerships where the HIV-infected partner is not on ART. Intensification of actions to prevent, detect and control STIs alongside HIV are important if the burden of STIs is to be reduced and would benefit from strengthened surveillance.

## Supporting information

S1 File Prevalence data and biomedical assumptions. Including:
A Table. Syphilis prevalence data from surveys, studies and routine ANC-based program screening, used in the Spectrum-STI estimation of adult syphilis prevalence in South Africa With references: [7, 8, 19, 46, 50, 70–97].
B Table. Spectrum assumptions on proportion of episodes symptomatic, proportions of symptomatic episodes treated, episode durations for treated and untreated episodes, and the resulting treatment coverage-weighted average episode durations. With references: [10, 19, 38].
C Table. Gonorrhea and chlamydia prevalence data used, and adjustments for diagnostic test performance and missing high-risk populations, and for chlamydia for age, in the Spectrum-STI prevalence trend estimation for 15–49 year-old adults, South Africa. With references: [32, 46, 50, 51, 72, 77, 80, 81, 86, 91–93, 95, 98–116].
(XLSX)

S2 File Reported STI cases, and STI prevalences among women with Vaginal Discharge Syndrome, South Africa. (DOCX)
