# Supplementary material for: Adult gonorrhea, chlamydia and syphilis prevalence, incidence, treatment and syndromic case reporting in South Africa: Estimates using the Spectrum-STI model, 1990-2017
Source: PLoS One. 2018 Oct 15;13(10):e0205863. doi: 10.1371/journal.pone.0205863 (PMC6188893; doi:10.1371/journal.pone.0205863)
Supplement: S2 File — (DOCX) [file pone.0205863.s002.docx]

**S2 file. Reported STI cases, and STI prevalences among women with Vaginal Discharge Syndrome, South Africa**

Supplementary file to: *Adult gonorrhea, chlamydia and syphilis prevalence, incidence, treatment and syndromic case reporting in South Africa: estimates using the Spectrum-STI model, 1990-2017*, by Kularatne-RS, Korenromp-EL et al., version 26 Sept. 2018

S2 Table records the annual number of new cases of Male Urethritis Syndrome (MUS), male and female Genital Ulcer Syndrome (GUS), and Vaginal Discharge Syndrome (VDS). The MUS figures are the number of Male Urethritis Syndrome MUS cases reported nation-wide to the National Indicator Dataset (NIDS) of the District Health Information System (DHIS)(1) by all 4,489 primary healthcare facilities, including 818 mobile clinic. All clinics are required to report this information (National Department of Health’s District Health Management Information System Policy 2011). National case reporting for MUS commenced in 2001. Data on the other indicators are from sentinel sites; since 2005 NIDS has been collecting data from 264 sentinel primary healthcare facilities (1)

For all three syndromes the annual number or cases reported and the corresponding rates declined from 2005 to 2016, except for a slight increase in 2017 for MUS and for GUS in both men and women (S1 Fig). For MUS, the 2005-2016 decline was stronger in the national data than in the subset of sentinel sites in the NIDS sentinel system (S1 Fig).

**S2 Table. Reported new STI diagnoses, by data system**

|  | **Male Urethritis Syndrome** | | **Genital Ulcer Syndrome** | | **Vaginal discharge syndrome** |
| --- | --- | --- | --- | --- | --- |
| **Year** | **Male** | | **Male** | **Female** | **Female** |
| **Data system** | **NIDS national**** | **NIDS sentinel*** | **NIDS sentinel*** | | **NIDS sentinel*** |
| 2001 | 482,382 |  |  |  |  |
| 2002 | 464,714 |  |  |  |  |
| 2003 | 432,590 |  |  |  |  |
| 2004 | 439,867 |  |  |  |  |
| 2005 | 420,582 | 32,093 | 4,944 | 5,105 | 52,404 |
| 2006 | 404,001 | 29,601 | 4,134 | 4,261 | 43,788 |
| 2007 | 365,819 | 25,846 | 3,001 | 2,996 | 35,738 |
| 2008 | 390,539 | 27,502 | 2,865 | 2,988 | 38,198 |
| 2009 | 396,745 | 26,484 | 2,804 | 2,929 | 36,612 |
| 2010 | 311,108 | 20,671 | 2,273 | 2,200 | 30,310 |
| 2011 | 239,949 | 12,093 | 2,265 | 1,932 | 24,983 |
| 2012 | 267,687 | 14,899 | 1,998 | 1,776 | 18,577 |
| 2013 | 308,830 | 16,609 | 1,349 | 1,538 | 17,841 |
| 2014 | 338,586 | 20,458 | 1,379 | 1,324 | 18,637 |
| 2015 | 320,029 | 19,665 | 750 | 804 | 13,651 |
| 2016 | 270,827 | 17,784 | 488 | 529 | 10,582 |
| 2017 | 310, 921 | 19,182 | 643 | 894 | 10,473 |
| 2001 denominator population (15-49 years) | 11,631,353 |  |  |  |  |
| 2005 denominator population (15-49 years) | 12,371,145 | 796,872 | 796,872 | 743,393 | 743,393 |
| 2017 denominator population (15-49 years) | 15,290,995 | 703,411 | 703,411 | 627,296 | 627,296 |

* NIDS sentinel = MUS, GUS and VDS case reporting to NIDS from sentinel PHC sites only; denominator population is the catchment population of these PHCs.

** NIDS national = MUS case reporting to NIDS from all national primary healthcare facilities; denominator population is South Africa’s national 15-49 years population.

Sources: (1).

**S1 Fig. STI syndrome reporting rates, in South Africa’s sentinel and national health information system**

Notes to S1 Fig: Data (numerators and denominators) from Table S2 above.

* MUS (NIDS sentinel), Genital Ulcer Syndrome (men and women) and Vaginal Discharge Syndrome = MUS, GUS and VDS case reporting to NIDS from sentinel PHC sites only; denominator population is the catchment population of these PHCs.

** MUS (national) = MUS case reporting to NIDS from all national primary healthcare facilities; denominator population is South Africa’s national 15-49 years population.

**S2 Fig. Gonorrhea, chlamydia, trichomonas vaginalis and *Mycoplasma genitalium* prevalences among women with Vaginal Discharge Syndrome**

Note to S2 Fig: VDS definition: women aged 18 years and above presenting with abnormal vaginal discharge, to sentinel primary healthcare facilities included in NICD GERMS-SA STI surveillance (The number of sites sampled varies by year, with never more than 1 site per province: Gauteng Province 2007 – 2014; Northern Cape 2006 & 2012; Western Cape 2006; Free State 2007; Limpopo & North-West Provinces 2011; Mpumalanga & Kwa-Zulu Natal Provinces – 2013 (2, 3)). The gonorrhea and chlamydia prevalences shown here were added as data points, complementing the basic/default data shown in S1C Table, only in a Sensitivity analysis (4^th^ scenario after the Best/default, in Table 3). Aetiologies measured through NAAT (specifically, a validated in-house real-time multiplex PCR assay) on endocervical swab; the prevalence data shown have not been adjusted for diagnostic test. Source: (1).

**References for S2 file:**

1. South Africa National Department of Health. National Integrated District Health Information System Pretoria [cited 2018 07 June]. Available from: <https://za.dhis.dhmis.org/dhis-web-commons/security/login.action>.

2. Mhlongo S, Magooa P, Muller EE, Nel N, Radebe F, Wasserman E, et al. Etiology and STI/HIV coinfections among patients with urethral and vaginal discharge syndromes in South Africa. Sex Transm Dis. 2010;37(9):566-70.

3. Kularatne R. Aetiological surveillance of sexually transmitted infection syndromes at sentinel sites: GERMS-SA 2014-2016. Public Health Surveillance Bulletin. 2017;15:114-22. http://www.nicd.ac.za/wp-content/uploads/2017/03/NICD-Bulletin-Vol_15_Iss3-November_2017.pdf
